# Analysis of the Immune Responses in the Ileum of Gnotobiotic Pigs Infected with the Recombinant GII.p12_GII.3 Human Norovirus by mRNA Sequencing

**DOI:** 10.3390/v13010092

**Published:** 2021-01-11

**Authors:** Byung-Joo Park, Hee-Seop Ahn, Sang-Hoon Han, Hyeon-Jeong Go, Dong-Hwi Kim, Changsun Choi, Soontag Jung, Jinjong Myoung, Joong-Bok Lee, Seung-Yong Park, Chang-Seon Song, Sang-Won Lee, Hoon-Taek Lee, In-Soo Choi

**Affiliations:** 1 Department of Infectious Diseases, College of Veterinary Medicine, Konkuk University, Gwangjin-gu, Seoul 05029, Korea; twilightsd@naver.com (B.-J.P.); heesuob2@naver.com (H.-S.A.); hansh11@naver.com (S.-H.H.); misilseju@naver.com (H.-J.G.); opeean0@naver.com (D.-H.K.); virus@konkuk.ac.kr (J.-B.L.); paseyo@konkuk.ac.kr (S.-Y.P.); songcs@konkuk.ac.kr (C.-S.S.); odssey@konkuk.ac.kr (S.-W.L.); 2 Department of Food and Nutrition, College of Biotechnology and Natural Resources, Chung-Ang University, Anseong, Gyeonggi 17546, Korea; cchoi@cau.ac.kr (C.C.); amazing2257@gmail.com (S.J.); 3 Korea Zoonosis Research Institute, Chonbuk National University, Jeonju, Jeollabuk-do 54896, Korea; jinjong.myoung@jbnu.ac.kr; 4 Department of Bioscience and Biotechnology, Konkuk University, Seoul 05029, Korea; htl3675@konkuk.ac.kr

**Keywords:** human norovirus, gnotobiotic pig, immune response, high-throughput mRNA sequencing

## Abstract

Norovirus genogroup II (NoV GII) induces acute gastrointestinal food-borne illness in humans. Because gnotobiotic pigs can be infected with human norovirus (HuNoV) GII, they are frequently used to analyze the associated pathogenic mechanisms and immune responses, which remain poorly understood. Recently, mRNA sequencing analysis (RNA-Seq) has been used to identify cellular responses to viruses. In this study, we investigated the host immune response and possible mechanisms involved in virus evasion in the ileum of gnotobiotic pigs infected with HuNoV by RNA-Seq. HuNoV was detected in the feces, blood, and tissues of the jejunum, ileum, colon, mesenteric lymph node, and spleen of pigs infected with HuNoV. In analysis of mRNA sequencing, expression of anti-viral protein genes such as OAS1, MX1, and MX2 were largely decreased, whereas type I IFN was increased in pigs infected with HuNoV. In addition, expression of *TNF* and associated anti-inflammatory cytokine genes such as *IL10* was increased in HuNoV-infected pigs. Expression of genes related to natural killer (NK) cell cytotoxicity and CD8^+^ T cell exhaustion was increased, whereas that of MHC class I genes was decreased. Expression profiles of selected genes were further confirmed by qRT-PCR and Western blot. These results suggest that infection with HuNoV induces NK cell-mediated cytotoxicity but suppresses type I IFN- and CD8^+^ T cell-mediated antiviral responses.

## 1. Introduction

Norovirus (NoV) constitutes an important pathogen of food-borne illnesses in humans. Human norovirus (HuNoV) is transmitted by the fecal–oral route and generally causes self-limiting acute gastroenteritis characterized by diarrhea, vomiting, stomach pain, and fever [1]. HuNoV infection leads to approximately 20 million illnesses, 60,000 hospitalizations, and 700 deaths annually in the United States [2,3]. NoVs comprise non-enveloped single-stranded positive-sense RNA viruses belonging to the family *Caliciviridae* [1]. They are putatively classified into ten genogroups and further divided into at least 49 genotypes [4], with NoVs of genogroups I (GI), II (GII), and IV (GIV) representing the main causative agents of most human infections [5].

However, although the clinical features of NoV gastroenteritis are well established, the target cells of HuNoV infection and associated host immune responses are poorly understood because of previous difficulties in identifying suitable cell culture systems and establishing animal models [6]. Initially, human B cells were proposed to constitute a major target of HuNoV, with reproductive viral replication being demonstrated in these cells [7]. Human enterocytes, macrophages, T cells, and dendritic cells (DCs) of the small intestine have also been suggested as HuNoV targets [8]. In addition, the production of T helper type 1 (Th1) cytokines and acute chemokines is observed in patients infected with HuNoV [9].

Gnotobiotic pigs have been used as an alternative animal model to study the viral pathogenesis and immune responses to HuNoV infection because they harbor the histo-blood group of antigens shown to function as the receptor for the virus [10]. Newborn gnotobiotic pigs infected with HuNoV demonstrate mild diarrhea, virus replication, and histopathologic lesions in the small intestinal tract [11]. Post-weaning gnotobiotic pigs are also used to study viral pathogenesis of HuNoV infection and target tissues of HuNoV in the pig [12,13]. Those studies identified experimental infectious doses 50 of HuNoV, such as 2.74 × 10^3^ and 6.43 × 10^4^ genomic equivalent (GE) copies in neonatal piglets and 33–34 days old pigs, respectively. They also found that the administration of simvastatin into pigs produced the increased infectivity of HuNoV. Notably, gnotobiotic pigs infected with HuNoV produce high levels of Th1 cytokines, albeit low levels of Th2 and proinflammatory cytokines, similar to that observed in human patients [9,14]. Additionally, a longer period of viral shedding is exhibited in RAG2/IL2RG-deficient pigs, indicating the importance of lymphocyte-mediated adaptive immune responses for elimination of HuNoV [15].

Understanding of NoV viral pathogenesis and immune responses has been expanded since the discovery of murine norovirus (MuNoV) [16]. Whereas HuNoV only induces acute infection in immunocompetent individuals, different MuNoV strains may cause persistent or acute infection in wild-type mice [17,18]. MuNoV induces systemic infection, which leads to dissemination of the virus to various tissues, such as the intestine, spleen, liver, and lung [19]. Moreover, infection by MuNoV has been identified in intestinal macrophages, DCs, B lymphocytes, and T lymphocytes as well [16,20,21,22]. Although wild-type mice infected with MNV do not experience diarrhea, severe diarrhea is induced in interferon-deficient mice [19]. MNV infection is also controlled by the innate and adaptive immune systems [23,24,25]. Recently, a gene expression profiling study was conducted via a high-throughput mRNA sequencing (RNA-Seq) method to determine up- and downregulated genes in MuNoV-infected mouse macrophage cell lines [26]. However, it remains unclear how the innate and adaptive immune systems are induced or suppressed in the small intestine during the acute period of HuNoV or MuNoV infection.

In this study, we ascertained the comprehensive immune responses induced in the small intestine of gnotobiotic pigs that had been infected with HuNoV. The mRNA expression profiles of the ileum were investigated by using high-throughput sequencing and confirmed by quantitative reverse-transcription-PCR (qRT-PCR) and Western blotting analyses. Generally, infection with HuNoV appeared to induce the activation of natural killer (NK) cells and suppression of CD8^+^ T lymphocytes. This new information, provided in this study, will substantively contribute to an improved understanding of NoV pathogenesis and immunology.

## 2. Materials and Methods

### 2.1. Virus Preparation

HuNoV strain CAU140599 (GenBank accession no. MN199033), HNV GII.p12_GII.3, obtained from a patient suffering from acute gastroenteritis, was used as the infectious agent [27,28]. The virus stock was diluted 10 fold with minimum essential medium (Wellgene, Gyeongsan, Korea). The diluted solutions were centrifuged at 3000× *g* for 30 min and the supernatant was collected. RNA was extracted from the supernatant using a QIAamp viral RNA extraction kit (Qiagen, Valencia, CA, USA), and the viral RNA was stored at −70 °C.

### 2.2. Animals and Experimental Design

A total of 15, four-week-old post-weaning gnotobiotic pigs were provided by the Bio-organ Research Center, Konkuk University, Seoul, Korea. The experimental procedures were approved by the Institutional Animal Care and Use Committee (IACUC), Konkuk University, Korea (approval number KU14073). The pigs were acclimatized for 3 days at a biosecurity level 2 grade facility by providing commercial feed prior to conducting experiments. All infection experiments were performed at the biosecurity level 2 grade-facility located at the College of Veterinary Medicine, Konkuk University. Pigs were divided into two experimental groups: a mock-infected negative control group and a HuNoV-infected group. Five pigs were used as negative controls. Ten pigs were infected with 1 × 10^7^ genome equivalent (GE) copies of HuNoV. Blood and rectal swab samples were collected every day for 3 days after viral infection. All pigs were euthanized at day 3 with potassium chloride after administering zolazepam. Gross pathogenic legions of the small and large intestines, mesenteric lymph nodes, and spleen were examined. The collected organs were stored at –70 °C and fixed in 4% formaldehyde.

### 2.3. Preparation of Fecal and Serum Samples

The rectal swab samples collected from the gnotobiotic pigs were diluted 1:10 (*w/v*) with phosphate-buffered saline (PBS). The fecal samples were placed in 1.5 mL microcentrifuge tubes and 500 μL of PBS was added. All the diluted samples were centrifuged at 3000× *g* for 30 min and supernatants were collected. The blood samples were collected from the jugular veins of the gnotobiotic pigs and the serum samples were separated by centrifugation at 2000× *g* for 10 min. Viral RNA was extracted from the supernatants of the fecal and serum samples using a QIAamp viral RNA extraction kit (Qiagen, Hilden, Germany).

### 2.4. Detection of NoV in Fecal, Serum, and Tissue Samples

Semi-nested qRT-PCR was conducted using a T100™ Thermal Cycler (BioRad, Hercules, CA, USA) to detect NoV in the fecal and serum samples. The first qRT-PCR was conducted with a total volume of 20 μL consisting of 10 pmol of each GII-forward 1 (GII-F1) and GII-reverse 1 (GII-R1) primer, 5 μL of RNA sample, and a Maxime RT-PCR premix kit (iNtRON Biotechnology, Seoul, Korea) (Table S1). The first qRT-PCR conditions were: 45 °C for 30 min; 94 °C for 5 min; 25 cycles of 95 °C for 30 s, 50 °C for 30 s, and 72 °C for 30 s; and a final extension at 72 °C for 10 min. The second PCR was conducted using 2 μl of the first PCR product and GII-F3 and GII-R1 primers in the Maxime PCR premix kit (i-star taq) (iNtRON Biotechnology); the amplification reaction was expected to produce a 310 bp product (Table S1). The second PCR conditions were: 94 °C for 5 min; 25 cycles of 95 °C for 30 s, 50 °C for 30 s, and 72 °C for 30 s; and a final extension at 72 °C for 10 min. For detection of NS vRNA, cDNA was synthesized as follows: 10 μL of RNA mixture was prepared with 10 pmol of GII-F1 primer, 8 μL of RNA extracted from the spleen and mesenteric lymph node, and 1 μL of 10 mM dNTP mix. The RNA mixture was incubated at 65 °C for 5 min and then placed on ice for 1 min. The cDNA synthesis mixture was prepared by adding 2 μL of RT buffer to the RNA mixture, with 4 μL of 25 mM MgCl_2_, 2 μL of 0.1 M dithiothreitol, 1 μL of RNaseOUT, and 1 μL of SuperScript III RT (Invitrogen, Carlsbad, CA, USA). The cDNA synthesis mixture was incubated at 50 °C for 50 min and then at 85 °C for 5 min. Finally, 1 μL of RNase H was added and incubated at 37 °C for 20 min. The first PCR amplification was conducted using 5 μL of the cDNA and a GII-F1 and a GII-R1 primer pair in the Maxime PCR premix kit (i-star taq). The PCR conditions were: 94 °C for 5 min; 25 cycles of 95 °C for 30 s, 50 °C for 30 s, and 72 °C for 30 s; with a final extension at 72 °C for 10 min. The second PCR conditions were the same as described above.

### 2.5. Determination of Viral Dose and mRNA Expression Levels

qRT-PCR was used to calculate the viral dose in a 25 μL total reaction volume containing RNase-free water, 5 μL RNA sample, 12.5 μL of 2 × OneStep RT-PCR Buffer III (TaKaRa, Shiga, Japan), 0.5 μL of Ex Taq HS (TaKaRa), 10 pmol of each primer (QNIF2d and COG2R), and 5 pmol of probe (QNIFS) [29,30]. The qRT-PCR was performed using a SmartCycler II system (Cepheid, Sunnyvale, CA, USA) as follows: reverse transcription at 42 °C for 5 min; an initial denaturation at 95 °C for 10 s; a two-step amplification of 45 cycles; denaturation at 95 °C for 15 s; and annealing and extension at 60 °C for 30 s. The genomic equivalence of the virus was determined based on a standard curve constructed using synthetic HuNoV GII RNA (ATCC, Manassas, VA, USA). The mRNA was amplified by synthetic primer sets (Table S2).

### 2.6. RNA Isolation for High-Throughput mRNA-Sequencing (RNA-Seq)

Total RNA was isolated using TRIzol reagent (Invitrogen). RNA quality was assessed with an Agilent 2100 bioanalyzer using the RNA 6000 Nano Chip (Agilent Technologies, Amstelveen, The Netherlands), and RNA quantification was performed using the ND-2000 Spectrophotometer (Thermo Inc., Waltham, MA, USA).

### 2.7. Library Preparation and Sequencing

For control and test RNAs, the construction of the respective library was performed using the SENSE mRNA-Seq Library Prep Kit (Lexogen, Inc., Vienna, Austria) according to the manufacturer’s instructions. Briefly, 2 μg total RNA was prepared for each sample and incubated with magnetic beads decorated with oligo-dT; other RNAs except mRNA were removed with a washing solution. Library production was initiated by the random hybridization of starter/stopper heterodimers containing Illumina-compatible linker sequences to the poly(A) RNA still bound to the magnetic beads. A single-tube RT and ligation reaction extended the starter to the next hybridized heterodimer, where the newly synthesized cDNA insert was ligated to the stopper. Second strand synthesis was performed to release the library from the beads, and the library was then amplified. Barcodes were introduced when the library was amplified. High-throughput sequencing was performed as paired-end 100 sequencing using HiSeq 2500 (Illumina, San Diego, CA, USA).

### 2.8. Data Analysis

mRNA-Seq reads were mapped using the TopHat software tool in order to obtain the alignment file [31]. Differentially expressed genes were determined based on counts from unique and multiple alignments using coverage in Bedtools [32]. The RT (Read Count) data were processed based on a Quantile normalization method using EdgeR within R using Bioconductor [33,34]. The alignment files also were used for assembling transcripts, estimating their abundances, and detecting differential expression of genes or isoforms using cufflinks (http://cole-trapnell-lab.github.io/cufflinks/). We utilized fragments per kilobase of exon per million fragments (FPKM) as the method of determining the expression level of the gene regions. Gene classification was based on searches performed using DAVID (http://david.abcc.ncifcrf.gov/). The gene profile was analyzed using IPA version 49309495 (Qiagen, Hilden, Germany) [35]. The gene symbols and coding protein names are described in Table S3.

### 2.9. In Situ Hybridization for Histological Pathology and Western Blot Analysis

The jejunum, ileum, colon, spleen, mesenteric lymph nodes, and liver from each pig were placed in 4% neutral-buffered formaldehyde and embedded in paraffin using standard methods. Double-strand DNA probe was randomly labeled with digoxigenin (Roche, Madison, WI, USA) using purified nested RT-PCR product (Table S1). Tissue sections were deparaffinized, rehydrated, and digested with 0.2 N HCl and 100 µg/mL proteinase K for 20 min at 37 °C. After digestion, tissues were fixed with 4% paraformaldehyde for 10 min and washed with PBS. Subsequently, tissues were equilibrated for 10 min in 2× saline sodium citrate (SSC) (1× SSC contains 50 mM NaCl and 15 mM sodium citrate.) In situ hybridization assays on the tissue samples were performed as described previously [28].

Western blot analysis was performed for evaluation of protein expression as described previously [36]. We utilized antibodies against IFN-α (Cat no. 140889; United States Biologicals, Salem, MA, USA), MX1 (Cat no. ab79609; Abcam, Cambridge, UK), phosphorylated JAK1 (Cat no. MBS821826; OriGene, Rockville, MD, USA), TNF (Cat no. orb11495; Biorbyt, St. Louis, MO, USA), CTLA4 (Cat no. orb19589; Biorbyt), TGF-α (Cat no. LS-C352937-100; LifeSpan BioSciences, Seattle, WA, USA), NCR1 (Cat no. TA338174; OriGene), and GAPDH (Cat no. orb397248, Biorbyt). Quantification was performed using ImageJ software, following the manufacturer’s protocol for ImageJ Gel Analysis documentation.

## 3. Results

### 3.1. Identification of HuNoV Infection

The viral infectivity was determined by examining fecal viral shedding, viremia, and viral RNA in tissues. As expected, viral infection was not detected from negative control pigs (Table 1). However, viral shedding was identified in fecal samples of 70–100% of pigs infected with HuNoV over 3 days (Table 1). Viremia was also detected during the same period in 10–40% of pigs (Table 1).

**Figure 1:**
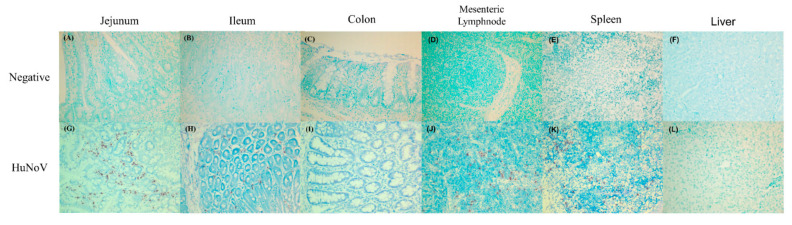
Detection of HuNoV RNA in tissues of gnotobiotic pigs by in situ hybridization. The viral RNA was determined by in situ hybridization for the (**A**–**F**) negative control pigs, (**G**–**L**) pigs infected with HuNoV. NoV-positive cells are stained as red colors in the cytoplasm of cells. Magnification ×200.

The presence of HuNoV RNA was confirmed by in situ hybridization in the jejunum, ileum, large intestine, mesenteric lymph node, spleen, and liver. The viral RNA was not observed in the liver samples of pigs infected with HuNoV (Figure 1L) and any examined tissues of negative control pigs (Table 1 and Figure 1A–F). However, viral RNA was identified in almost all of the samples of ileum (100%), jejunum (90%), large intestine (80%), mesenteric lymph node (100%), and spleen (100%) of pigs infected with HuNoV (Table 1).

### 3.2. Inflammation and IFN Immune Responses

A total of 26,965 genes were analyzed in the total RNA prepared from the ileum samples collected from all 15 experimental pigs by RNA-Seq. A total of 16,234 genes were mapped using Ingenuity Pathway Analysis (IPA) software, of which 5812 exhibiting over 1.5 fold change in expression were used for analysis.

**Figure 2:**
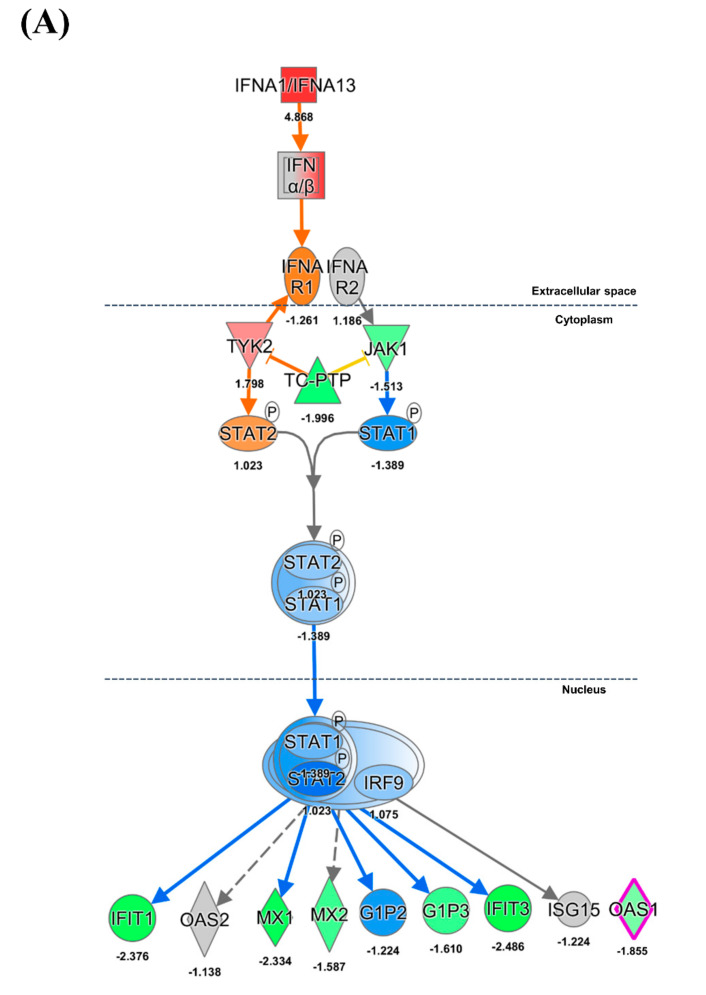

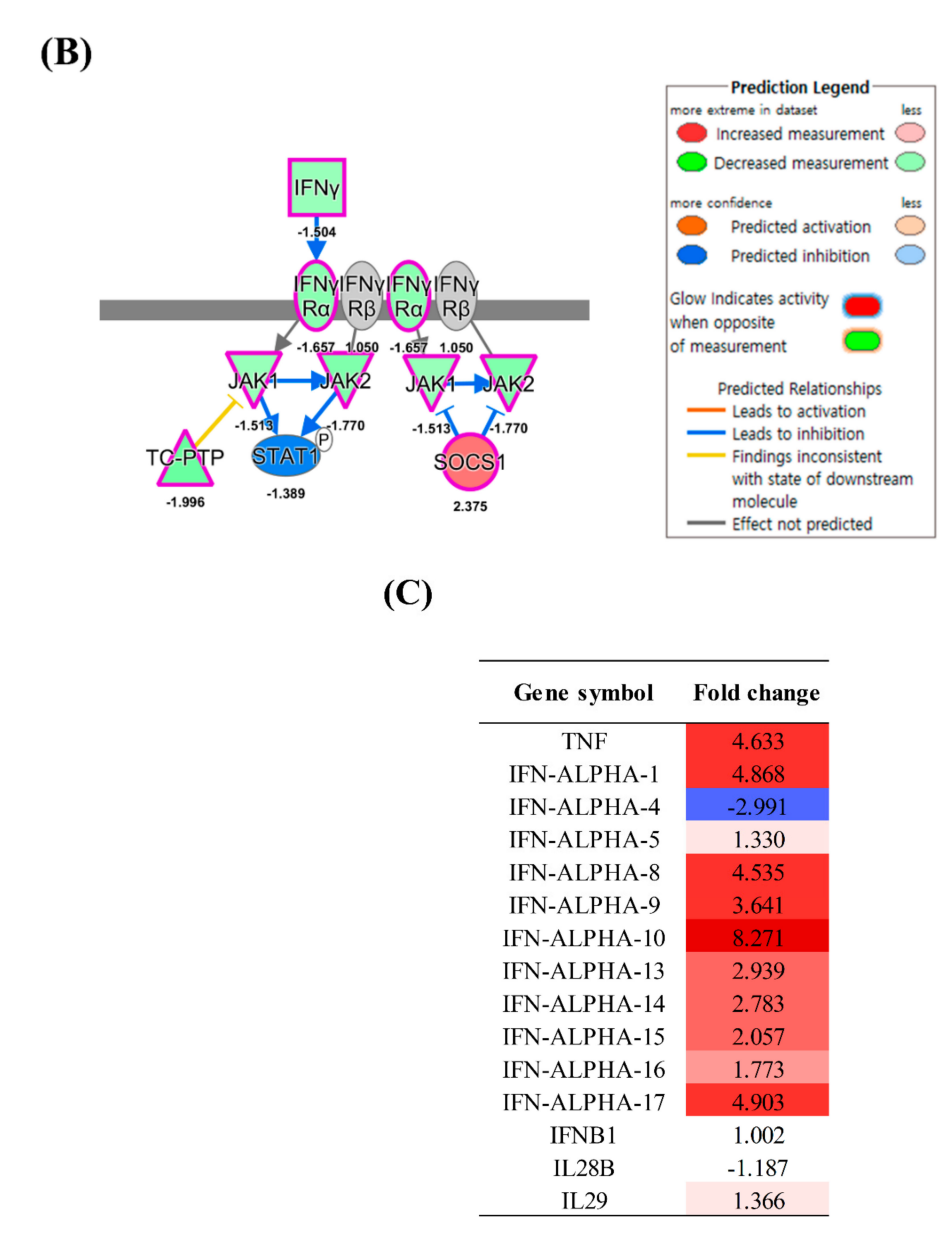
Ingenuity pathway analysis of type I and II interferon pathway genes in the HuNoV-infected group. The pathways of (**A**) type I IFN and (**B**) type II IFN were analyzed using mRNA profiles of pigs infected with HuNoV compared with those of negative control pigs. (**C**) Fold change expression values of *TNF, IFN-α*, *IFN-β*, and *IFN-λ* in pigs infected with HuNoV. The fold change of gene expression was indicated by colors in Table S4.

We evaluated inflammatory, innate, and adaptive immune responses in pigs that were infected with HuNoV. Gene expression changes in pigs infected with HuNoV were compared with those in negative control pigs. Almost all IFN-α subtypes were highly expressed at the mRNA level in the ileum of pigs infected with HuNoV (1.33–8.27 fold) (Figure 2A,C); however, the mRNA expression level of IFN-β1 (1.00 fold) did not change (Figure 2C). In addition, the expression of type III interferon genes (*IL28* and *IL29*) was also not changed (−1.18 and 1.36 fold, respectively, Figure 2C). Though the expression of genes encoding type I IFNs was upregulated, that of their receptor, *IFNAR1*, was slightly suppressed (−1.26 fold). The gene for TYK2, a mediator of the STAT complex, was highly expressed (1.79 fold), whereas expression of *JAK1* was inhibited (−1.51 fold). Anti-viral protein-coding genes such as *MX1* (−2.33 fold) and *OAS1* (−1.85 fold) were shown to be downregulated (Figure 2A).

Genes involved in the type II IFN signal pathway such as *IFNG* (−1.50 fold), *IFNGR1* (−1.65 fold), and *JAK1* and *JAK2* (each −1.77-fold) were downregulated (Figure 2B). IPA analysis suggested that increased mRNA expression of the suppressor of cytokine signaling 1 (*SOCS1*) (2.37 fold) would likely inhibit the phosphorylation of JAK1 and JAK2, ultimately leading to the inhibition of both type I and II IFN signaling pathways. High expression of *TNF-*α, the typical proinflammatory cytokine, was demonstrated (4.63 fold) in pigs infected with HuNoV (Figure 2C).

### 3.3. DC and NK Cell Activation

**Figure 3:**
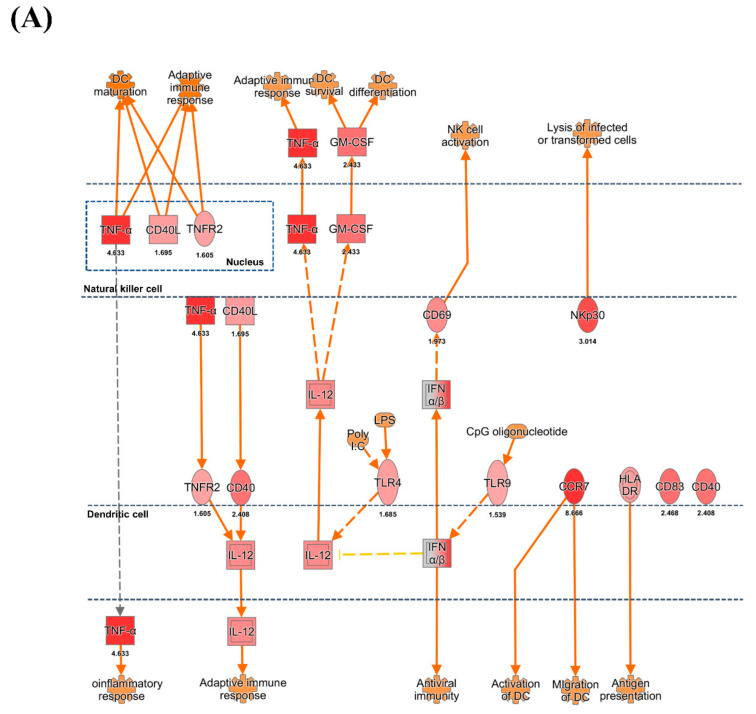

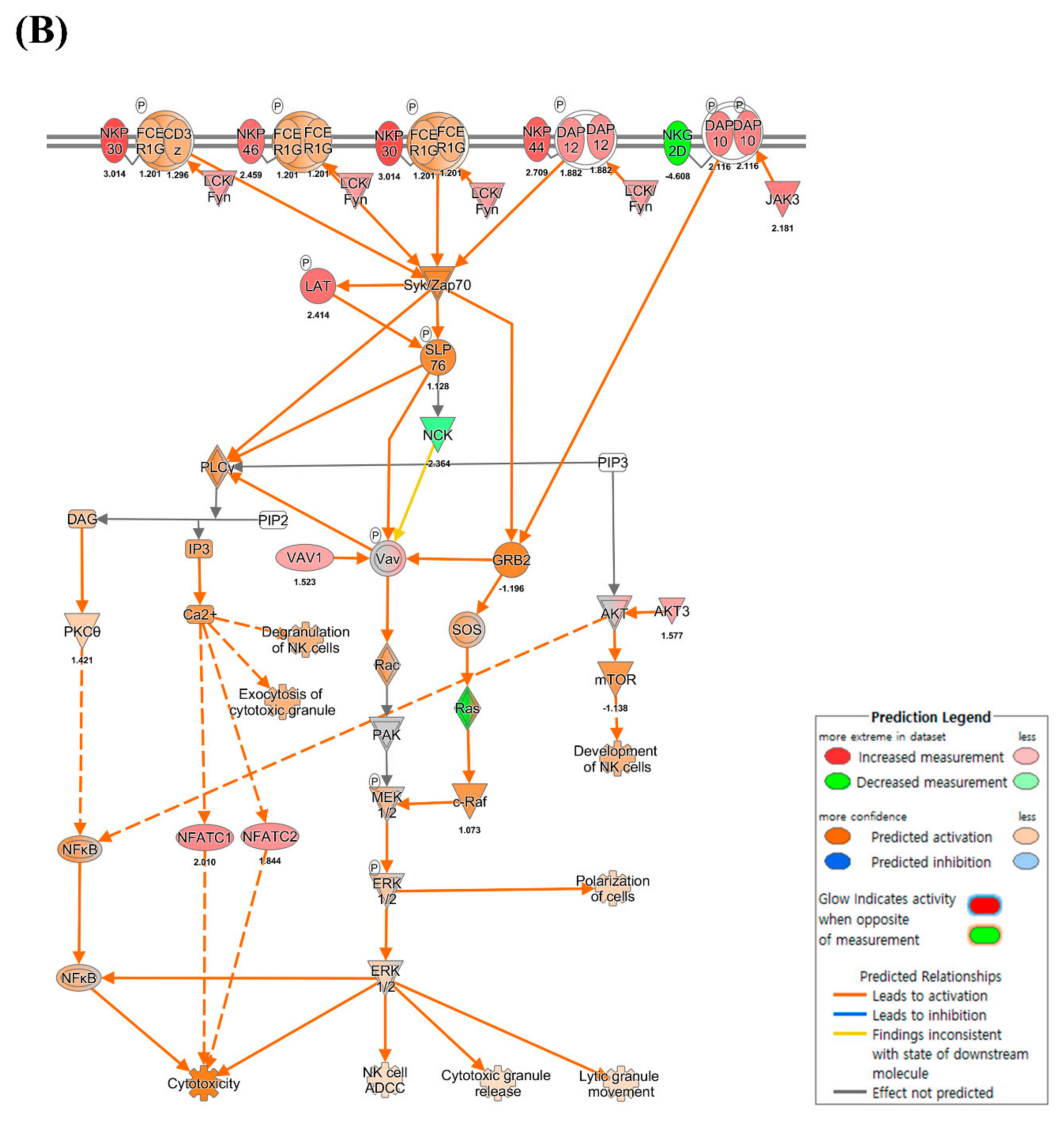

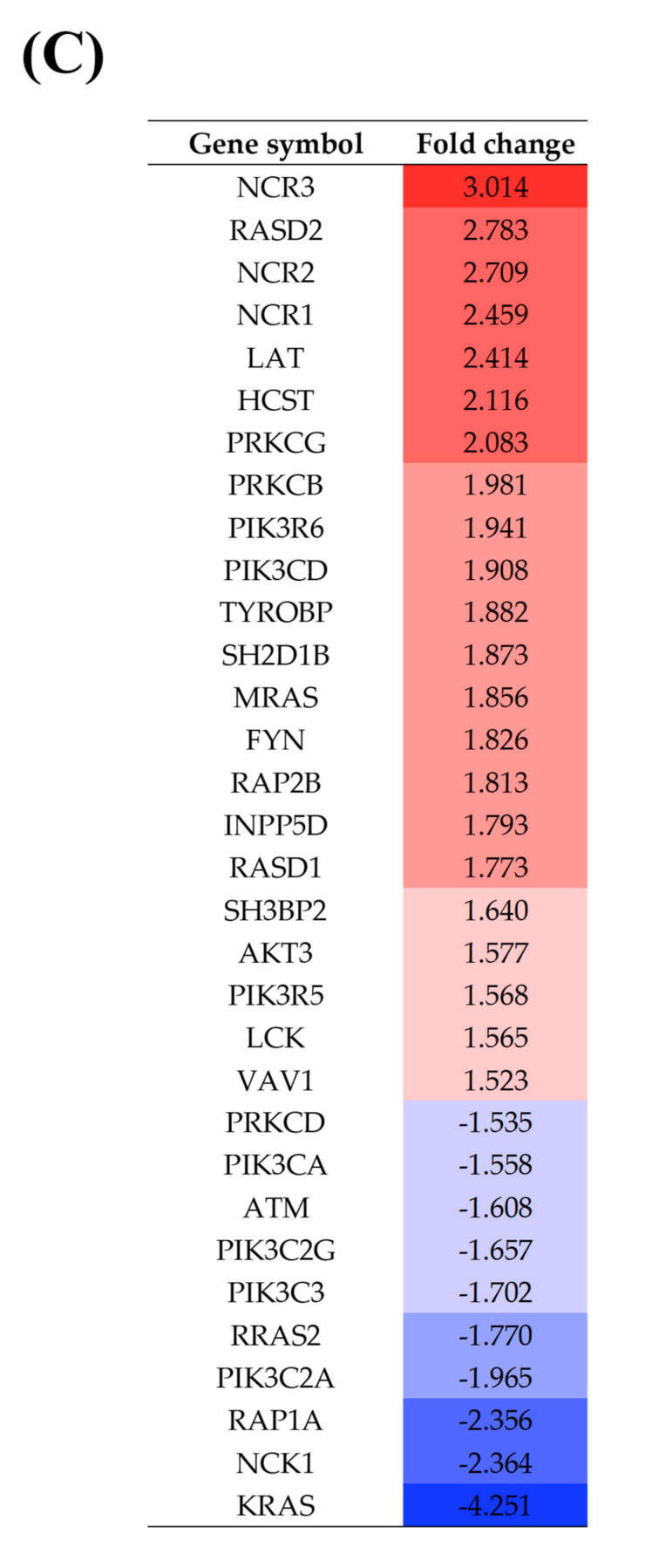
Ingenuity pathway analysis of genes associated with NK cell activation in the HuNoV-infected group. The (**A**) DC and NK cell interaction and (**B**) NK cell activation pathway were analyzed using mRNA profiles of pigs infected with HuNoV compared with those of negative control pigs. (**C**) Fold change expression values of NK cell-related genes in pigs infected with HuNoV. The fold change of gene expression was indicated by colors in Table S4.

Increased expression of *TLR4* (1.68 fold), *CD40* (2.40 fold), and *TNFRSF1B* (1.60 fold) was observed in pigs infected with HuNoV. Based on IPA, these gene expression profiles might lead to the increased expression of *IL12A* (ILp35) and *IL12B* (IL-12 p40) (2.02 and 1.97 fold, respectively) (Figure 3A). The high expression of *IL12A* and *IL12B* suggested the promotion of the T cell-mediated adaptive- and NK cell-mediated immune response. In addition, the increase of gene expression of TNF-α and CD40LG (1.69 fold) from NK cells appeared to promote maturation of DCs; moreover, the increased expression of *CCR7* (8.66 fold) suggested that the DCs might be activated. The genes for pattern recognition receptors (PRRs) such as *TLR4* (1.68 fold) and *TLR9* (1.53 fold), which induce expression of IFN-α, were also highly expressed on DCs. In turn, IFN-α might interact with the NK cell stimulatory receptor CD69 (1.97-fold), leading to NK cell activation.

In the NK cell pathway, NK-activating cytokine genes such as those encoding IL-12A/B and IFN-α were highly expressed on DCs. According to IPA, these expression patterns might lead to NK cell functional activation of three natural cytotoxicity receptors (NCRs), the expression of which was also elevated: *NKP46/NCR1* (2.45 fold), *NKP44/NCR2* (2.70 fold), and *NKP30/NCR3* (3.01 fold) (Figure 3B). The NK cell activation-mediator genes such as *DAP12*/*TYROBP* (1.88 fold), *LCK* (1.56 fold), *FYN* (1.82 fold), *LAT* (2.41 fold), *3BP2*/*SH3BP2* (1.64 fold), and *VAV1* (1.52 fold) were increased. Upregulation of these molecules might be connected with ERK1/2 signaling, which induces NK cell cytotoxicity. In contrast, the expression of NK inhibitory receptor genes such as *KIR* and *CD94*/*NKG2* was not changed.

### 3.4. CD4^+^ and CD8^+^ T Cell-Mediated Adaptive Immune Responses

**Figure 4:**
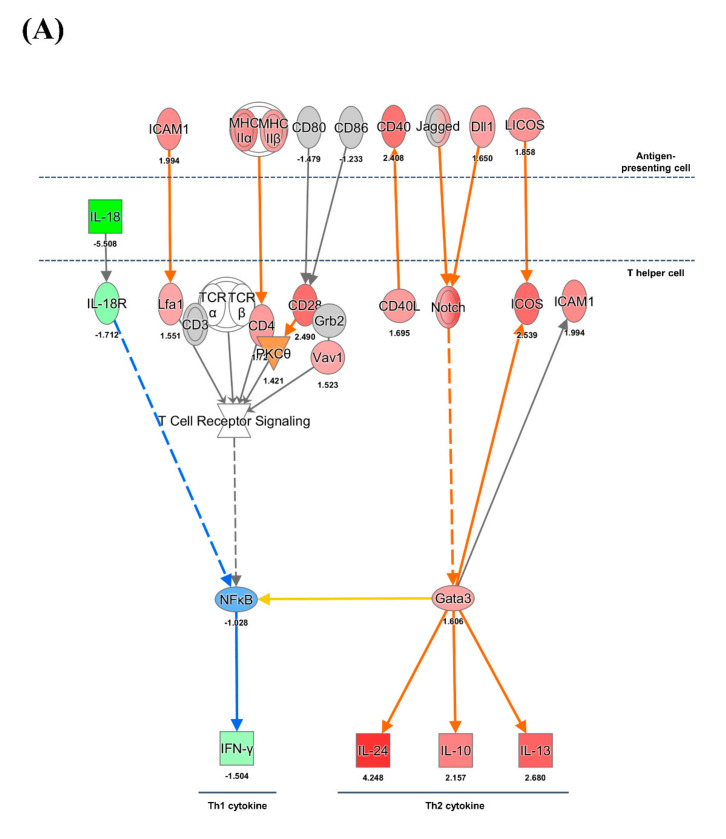

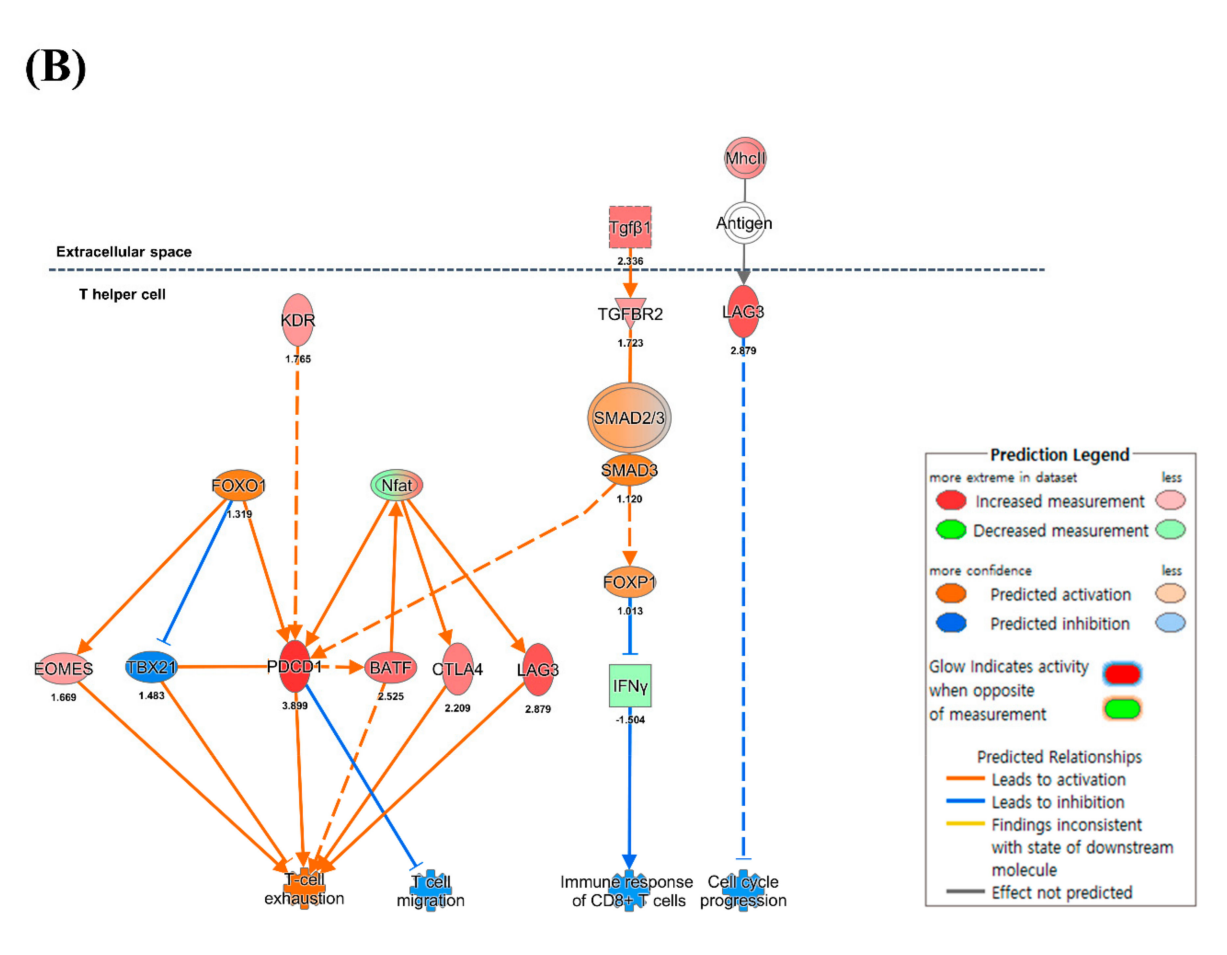

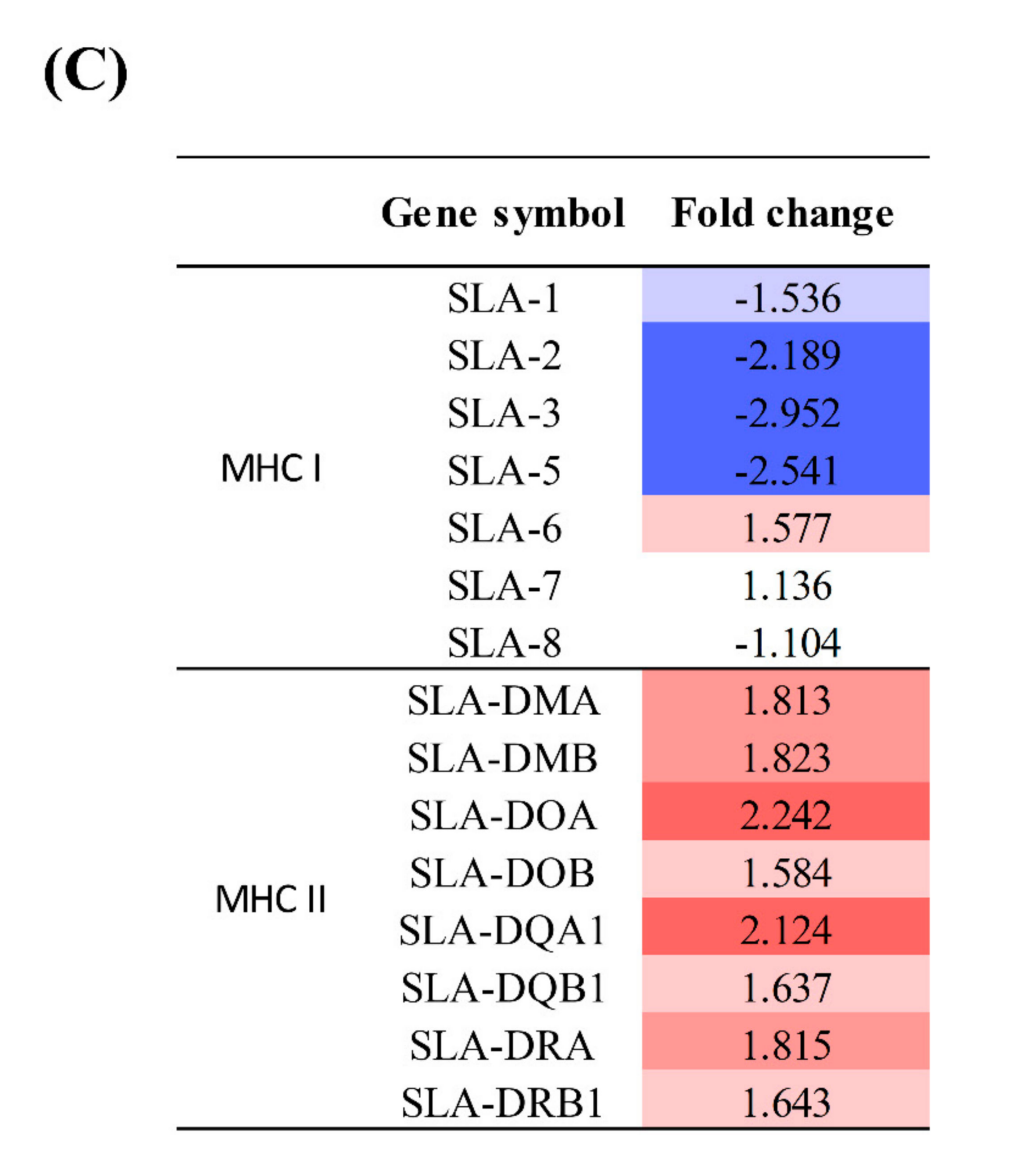
Ingenuity pathway analysis of genes associated with T cell immune response in the HuNoV-infected group. The (**A**) Th1/2 type immune response and (**B**) cytotoxic T cell exhaustion pathway were analyzed using mRNA profiles of pigs infected with HuNoV compared with those of negative control pigs. (**C**) Fold change expression values of *MHC I* and *II* in pigs infected with HuNoV. The fold change of gene expression was indicated by colors in Table S4.

Different expression patterns of MHC class I and II genes were identified in pigs infected with HuNoV. The gene expression of MHC class I group (−1.53 to −2.95 fold) was suppressed whereas MHC class II group genes (1.64–2.24 fold) were highly expressed (Figure 4A,C). According to IPA, these results suggested the potential for increased viral antigen presentation to CD4^+^ T cells through MHC class II albeit decreased viral antigen presentation to CD8^+^ T cells through MHC I. The co-stimulatory ligand-encoding genes such as *LICOS*/*ICOSLG* (1.85 fold), *ICAM1* (1.99 fold), Jagged/*JAG2* (1.90 fold), and *CD40* (2.40 fold) were highly upregulated on the antigen-presenting cells. The co-stimulatory receptor-encoding genes such as *ICOS* (2.53 fold), and Notch (1.54–3.45 fold) were upregulated on the CD4^+^ T cells. The gene for the GATA3 transcription factor (1.65-fold) was highly expressed and its activation might lead to expression of genes encoding Th2-type cytokines such as *IL10* (2.11 fold), *IL13* (2.68 fold), and *IL24* (4.24 fold). The IL-10 produced from CD4^+^ T cells would lead to suppression of IFN-γ from CD4^+^ T cells. Decreased expression of the *IL18* gene was identified from DCs and the *IL18R* gene on CD4^+^ T cells was also downregulated. The suppression of IL-18 appeared to inhibit the production of IFN-γ. The genes associated with T cell exhaustion such as *CTLA4* (2.20 fold), *BATF* (2.52 fold), *NFATC1* (2.0 fold), *NFATC2* (1.84 fold), *EOMES* (1.66 fold), *PDCD1* (3.89 fold), and *LAG3* (2.87 fold) were upregulated (Figure 4B).

### 3.5. Evaluation of mRNA Sequencing Data by qRT-PCR and Western Blot

**Figure 5:**
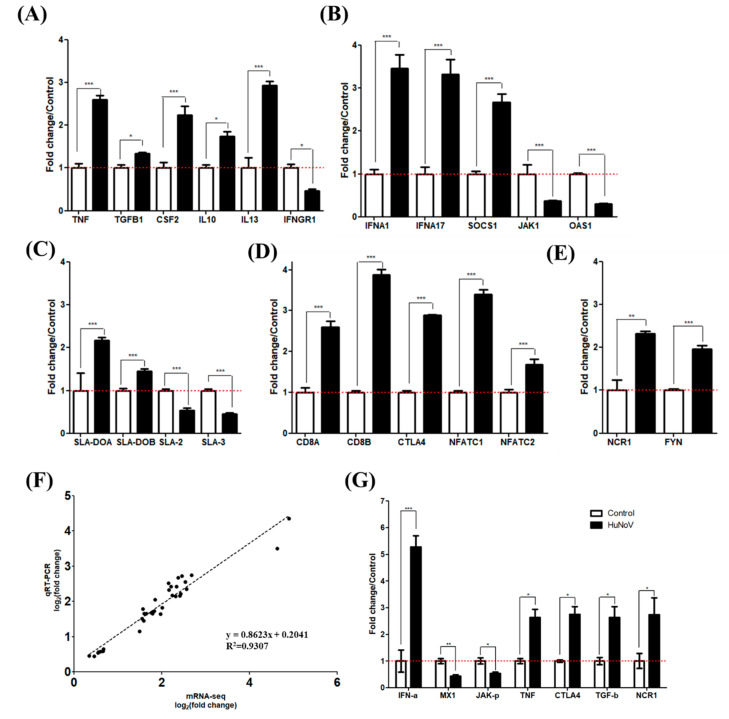
Evaluation of mRNA sequencing data by qRT-PCR and Western blot. The expression levels of genes associated with (**A**) cytokines, (**B**) innate immune response, (**C**) MHC molecules, (**D**) CD8^+^ cell exhaustion, and (**E**) NK cell activation in HuNoV-infected pigs (black) were compared with those of negative control pigs (white). (**F**) Correlation graph and trend line of evaluated genes between mRNA sequencing and qRT-PCR. The trend line expression was y = 0.8623x + 0.2041 and the R-squared value was 0.93. (**G**) Quantitation of Western blot analysis was performed using ImageJ. *** *p* < 0.001, ** *p* < 0.01, * *p* < 0.05.

We selected and determined the expression levels of representative genes associated with inflammation, innate immune response, NK cell activation, and adaptive immune response by qRT-PCR as follows (with fold-change by RNA-Seq): *TNF* (3.49 fold), *IFNA1* (1.65 fold), *IFNA17* (4.35 fold), *SOCS1* (2.67 fold), *JAK1* (−1.52 fold), *OAS1* (−1.83 fold), *NCR1* (2.72 fold), *FYN* (1.73 fold), *SLA*-*DOA* (2.17 fold), *SLA*-*DOB* (1.45 fold), *SLA2* (−2.27 fold), *SLA3* (−2.19-fold), *ICOS* (2.55 fold), *CD8A* (1.65 fold), *CTLA4* (2.42 fold), *NFATC1* (1.81 fold), and *NFATC2* (2.05 fold). All gene expression profiles re-examined with qRT-PCR exhibited a slope of 0.86 for the trend line and 0.93 for the R-squared value (Figure 5A–F). We also determined protein expression patterns of IFN-α, MX1, phosphorylated-JAK1, TNF, CTLA4, TGF-β, and NCR1 by Western blot and found that the expression patterns of proteins were similar to those exhibited by the encoding genes (Figure 5G and Figure S1).

## 4. Discussion

### 4.1. Pathogenesis of HuNoV

In the present study, the onset of viral shedding occurred at 1 day post-infection (dpi) and lasted for 2 to 3 days, and viremia occurred in 50% of HuNoV-infected gnotobiotic pigs, comparable to the results of other studies using pigs [11,12]. These results are also similar to those observed in human cases, in which HuNoV shedding begins at 1–2 dpi [37]. Rates of viremia determined in our study were also similar to those reported in another study, at approximately 56% [12]. Detection of viral RNA was also confirmed in almost all organs examined in the present study, such as the small intestine, large intestine, mesenteric lymph node, and spleen, except the liver.

MNV has been identified in macrophages, DCs, B cells, and T cells [7,38]. In comparison, HuNoV proteins have been identified in small intestinal epithelial cells [11,12]. In recent studies, B-derived cells were considered as potential target cells, and virus antigens were detected in the mesenteric lymph nodes and spleen in gnotobiotic pigs infected with HuNoV [7,13,28]. These results suggested that the intestine and immune organs are also involved in infection by HuNoV, similar to MNV.

### 4.2. Inflammatory Response

In early viral infection, TNF-α serves as the primary inflammatory molecule. TNF-α concentrations in the serum and fecal samples were dose-dependently correlated with severe clinical symptoms of humans and pigs infected with HuNoV [9,39,40]. In the present study, expression of TNF-α increased more than four-fold in gnotobiotic pigs infected with HuNoV. These results indicated that the inflammatory response was active in these pigs. In comparison, IL-10 plays an important role in the anti-inflammatory response during MNV infection because it prevents the mucosal inflammation induced by TNF-α [41]. In addition, the increased expression of IL-13 and TGF-β can exert anti-inflammatory effects through the stimulation of regulatory T cells. In our study, gene expression levels of IL-10, IL-13, and TGF-β were increased. Therefore, these data suggested that an initial inflammation induced by TNF-α upon HuNoV infection could be suppressed by subsequent induction of IL-10, IL-13, and TGF-β. These changes of cytokines after infection with the virus seem to be balanced between Th1 and Th2 cytokines.

### 4.3. Innate Immune Response

TLR4 and TLR9 have been shown to be stimulated by bacterial lipopolysaccharide and CpG motifs, respectively [42,43]. However, it has been reported that single-stranded RNA viruses such as dengue virus and Hantaan virus can induce TLR4/9 expression, leading to the induction of type I IFNs [44,45]. Furthermore, type I IFNs play an important role for clearance of MNV infection [24]. In the present study, expression of TLR4/9 and type I IFN genes was increased in gnotobiotic pigs infected with HuNoV. However, the expression of genes encoding JAK/STAT molecules and anti-viral genes such as *MX1* and *OAS1* were severely suppressed, which was accompanied by high expression of *SOCS1*. Increased expression of SOCS1 may suppress phosphorylation and expression of JAK [46,47]. In addition, the suppression of genes encoding type II IFNs and their downstream pathway (JAK/STAT) was identified in the present study. Both type I and II IFNs are important for resistance to MNV infection, as shown by the MNV lethality in mice lacking genes for both type I and II IFNs [16]. These results suggested that HuNoV effectively inhibits the expression of type II IFN and antiviral proteins by inhibiting the JAK/STAT pathway rather than by inhibiting expression of genes encoding type I IFNs. Previously, it has been reported that type III IFN is important for protection against MNV infection [18,48]. These results implied that type III IFN may play a critical role in clearance of MNV. In contrast, HuNoV infection in pigs did not induce the expression of type III IFN in our study. Further research may therefore be needed to understand the mechanism underlying the different expression patterns of type III IFN in mice and pigs infected with MNV and HuNoV, respectively.

### 4.4. NK Cell Activation

NK cells exhibit an inclination to kill tumor cells exhibiting suppressed expression of MHC molecules [49]. The cytotoxicity of NK cells occurs consequent to the absence of inhibitory signals recognized by the killer inhibitory receptor and by low expression level of MHC class I on virus-infected cells [50,51,52,53]. It has also been reported that NK cells cause cytotoxicity to cells infected with viruses such as retrovirus, herpesvirus, and hepatitis C virus [50,51,52,54]. Notably, high incidences of HuNoV infection were reported in patients with severe combined immunodeficiency (SCID); fecal shedding of HuNoV was significantly lower NK^+^ SCID patients compared with NK^-^ SCID patients [55]. However, a significant difference in HuNoV infections was not detected between NK^+^ SCID and non-SCID gnotobiotic pigs; in contrast, infectivity of HuNoV was increased in NK^-^ SCID pigs [15,56]. In these studies, the importance of NK cells in controlling HuNoV infection was suggested in human and SCID pigs [15,55,56]. In the present study, we observed suppression of MHC I gene expression in the pigs infected with HuNoV. Other researchers have shown that increased NCR expression levels of NK cells can effectively inhibit hepatitis C virus (HCV) infection [57,58]. The results of the present study further indicated that the IL-12 and IFN-α produced from DCs and T cells in pigs infected with HuNoV likely induced NK cell activation [59,60] followed by upregulation of NCR without activation of inhibitory receptor signaling. Collectively, both the high expression of genes encoding NK cell activation molecules and the suppression of those encoding MHC class I molecules implied the activation of NK cell cytotoxicity, which would be expected to induce cytotoxic effects toward HuNoV-infected cells. Therefore, our results and those of others indicate that NK cells may play important roles for the initial defense against HuNoV in pigs [15,55,56].

### 4.5. Expression of MHC Molecules and Cytokines

RNA and DNA viruses such as poliovirus and Epstein Barr Virus can suppress the expression of MHC class I molecules and the associated antigen presentation [61,62]. In contrast, no difference was detected in the expression levels of MHC class I and II molecules in mice infected with MNV, although the former have an important role in controlling acute infection in MNV infection [23,24]. MHC class I-deficient mice had shown prolonged MNV infection and significant levels of the viral titers in both the distal ileum and the MLN compared to WT mice [23]. In the present study, expression levels of MHC class I molecules were significantly decreased, whereas those of MHC class II molecules were increased. These results indicated that HuNoV infection induces suppression of CD8^+^ T cell-mediated cytotoxicity by inhibiting viral antigen presentation through MHC I molecules.

In other studies, Th1 (IL-12 and IFN-γ) and Th2 (IL-4 and IL-10) cytokines were initially induced in gnotobiotic pigs infected with HuNoV [9,14]. However, the level of IFN-γ rapidly decreased thereafter [14]. In the present study, the decreased expression of Th1-type cytokines (IFN-γ and IL-18) and the increased expression of Th2-type cytokines (IL-10, IL-13) was identified in pigs infected with HuNoV. Therefore, the results of our and other studies consistently indicated that HuNoV infection suppresses the Th1-type immune responses including IFN-γ. IL-10 has been shown to be involved in the development of B cells and production of antibodies [63,64]. In addition, as an anti-inflammatory cytokine, IL-10 induces downregulation of Th1 cytokine production. Therefore, these results clearly indicated that HuNoV elicits a strong Th2-type immune response but suppresses the Th-1-type immune response in pigs.

### 4.6. CD8^+^ T Cell Exhaustion

CD8^+^ T cell exhaustion has been reported in the chronic infections of human immunodeficiency virus, HCV, and lymphocytic choriomeningitis virus [65,66,67]. In addition, MNV-infected mice exhibited functional failure of the CD8+ T cell response, which resulted in persistent infection [68]. T cell exhaustion impairs cytokine production and cell proliferation during persistent infections [66,69,70]. It has been reported that PDCD1 (PD-1) and CTLA4 induce impairment of T cell functions in chronic infection [66,71,72,73]. In the present study, genes involved in CD8^+^ T cell exhaustion, such as *CTLA4*, *NFATC*, *EOMES*, *PDCD1*, *BATF*, and *LAG3*, were highly expressed. Therefore, high expression of these genes along with *IL10* and *IL13* may contribute to the suppression of T cell-mediated immune responses. In turn, increased expression of T cell exhaustion-associated genes along with decreased expression of MHC class I and Th1 cytokines implies that HuNoV infection inhibits the CD8^+^ T cell-mediated immune response. These results collectively indicated that clearance of HuNoV would not be achieved by CD8^+^ T cells but might be conducted by NK cells.

A limitation of this study was that gene and protein expression profiles were evaluated only in the ileum of pigs infected with a single recombinant strain of HuNoV. However, our results indicated that other tissues such as the large intestine, spleen, and mesenteric lymph nodes of pigs were also infected with HuNoV. Therefore, immune responses against HuNoV in the other tissues should be determined in future studies. In addition, single cell mRNA sequencing will provide more precise immune response information for each cell type in specific tissues infected with HuNoV [74,75,76,77].

## 5. Conclusions

**Figure 6:**
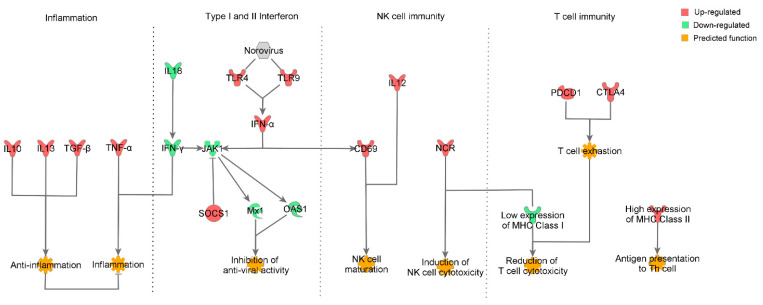
Summary of the immune response in pigs infected with HuNoV with regard to inflammation, type I and II interferon pathways, NK cells, and T cell immune response. Inhibition of the expression of antiviral genes was observed, whereas the TLR and IFN-α genes were increased in the HuNoV-infected cells. Decreased expression of MHC I and IFN-γ genes and elevated expression of genes associated with T cell exhaustion strongly suggest that antiviral activity of cytotoxic T cells is suppressed by the viral infection. On the other hand, the elevation of NCR genes related to NK cell activation suggests that NK cells are critically involved in the control of norovirus in early period of infection.

In pigs infected with HuNoV, the gene expression of type I IFNs was increased, whereas that of the antiviral proteins and JAK was decreased. Expression of MHC class I genes was severely reduced, whereas expression of genes associated with T cell exhaustion was increased. However, the expression of genes associated with activation of NK cells was increased (Figure 6). Collectively, these results suggested that although HuNoV infection inhibits the CD8^+^ T cell-mediated immune responses, NK cells would be expected to play a critical role in the control of HuNoV infection in pigs. Thus, the data presented in this study clarifies the intestinal pathological mechanisms and immune response in norovirus-mediated viral gastroenteritis, thereby providing a fundamental information for the development of anti-viral agents specific to HuNoV and an effective NoV vaccine for humans.

## Figures and Tables

**Table 1:** Detection of human norovirus RNA from fecal, serum, and tissue samples in experimental pigs.

Group	dpi *	0	1	2	3	Jejunum	Ileum	Large Intestine	Mesenteric Lymph Node	Spleen	Liver
	No.	F/S ^a^	F/S	F/S	F/S						
Negative control	428	−/−	−/−	−/−	−/−	−	−	−	−	−	−
	429	−/−	−/−	−/−	−/−	−	−	−	−	−	−
	490	−/−	−/−	−/−	−/−	−	−	−	−	−	−
	404	−/−	−/−	−/−	−/−	−	−	−	−	−	−
	403	−/−	−/−	−/−	−/−	−	−	−	−	−	−
HuNoV	422	−/−	−/−	+/−	+/+	+	++	+	+++	+++	−
	423	−/−	+/+	+/+	+/−	++	+++	+	+++	+++	−
	424	−/−	+/−	+/+	+−	+	+++	+	+++	+++	−
	497	−/−	−/−	+/−	+/−	++	++	+	+++	++	−
	499	−/−	+/−	+/−	−/−	+	+++	+	+++	+	−
	425	−/−	+/−	+/−	−/−	+	++	−	+	+++	−
	426	−/−	+/−	+/−	+/−	+	++	+	+	+++	−
	427	−/−	+/−	+/+	+−	+	++	−	++	++	−
	498	−/−	−/−	+/−	+/−	−	+	+	+	+	−
	500	−/−	+/+	+/+	+−	+	+++	+	+++	++	−

* dpi: days post-infection; ^a^ F: feces, S: serum.

## Data Availability

The analyzed gene expression profile and raw sequencing data were deposited in GenBank under GEO accession no. GSE144073. The raw sequencing reads were submitted to the SRA under accession no. SRP243917 (BioProject no. PRJNA602689).

## Supplementary Materials

The following are available online at https://www.mdpi.com/1999-4915/13/1/92/s1, Figure S1: Western blot analysis of major proteins associated with immune response in experimental pigs infected with HuNoV and uninfected controls, Table S1: List of primer sets and probe used to detect HuNoV RNA, Table S2: List of genes and primer sets used to evaluate mRNA sequencing data by qRT-PCR, Table S3: List of genes used for mRNA sequencing data analysis, Table S4: The color scale index for representing the relative expression level. The gene expression levels were indicated by different colors ranging from blue (low) to red (high).
