# Supplementary material for: Analysis of the Immune Responses in the Ileum of Gnotobiotic Pigs Infected with the Recombinant GII.p12_GII.3 Human Norovirus by mRNA Sequencing
Source: Viruses. 2021 Jan 11;13(1):92. doi: 10.3390/v13010092 (PMC7826840; doi:10.3390/v13010092)
Supplement: Supplementary file 1 [file viruses-13-00092-s001.pdf]

Supplemental Material

Figure S1. Western blot analysis of major proteins associated with immune response in experimental pigs infected with HuNoV and uninfected controls. The major proteins were selected via pathway analysis as IFN-◎, MX1, phosphorylated JAK1(Jak-p), TNF, CTLA4, TGF-◎, NCR1, and GAPDH.

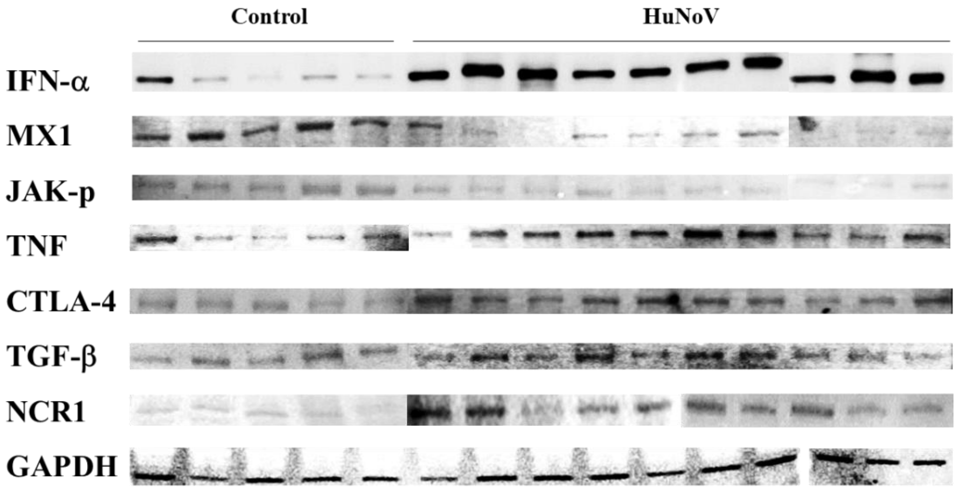

Table S1. List of primer sets and probe used to detect HuNoV RNA

Region	Primers	Sequence (5′-3′)	Polarity	Position[a]
ORF1-ORF2	GII-F1M	5′-GGG AGG GCG ATC GCA ATC T-3′	Forward	5049–5067
	GII-R1M	5′-CCR CCI GCA TRI CCR TTR TAC AT-3′	Reverse	5367–5389
	GII-F3M	5′-TTG TGA ATG AAG ATG GCG TCG ART-3′	Forward	5079-5102
	Probe	5'-TTG TGA ATG AAG ATG GCG TCG AGT GAC GCC ACC CCA TCT AAT GAT GGT GCC GCC GGC CTC GTC CCA GAG ATC AAC AAT GAG GCA ATG GCG CTA GAG CCA GTG GCG GGT GCA GCG ATA GCG GCA CCC CTC ACT GGC CAG CAA AAT ATA ATT GAT CCC TGG ATT ATG AAT AAT TTT GTG CAA GCA CCT GGT GGT GAG TTC ACA GTG TCT CCT AGG AAT TCC CCT GGT GAA GTG CTC CTC AAT TTG GAA TTG GGC CCA GAA ATA AAC CCC TAT CTG GCC CAT CTT GCT AGA ATG TAC AAT GGC TAT GCC GGT GG-3'	Forward	5079-5389

[a] Positions in a reference strain (GenBank accession number X86557)

Table S2. List of genes and primer sets used to evaluate mRNA sequencing data by qRT-PCR

Gene name	Forward Primer	Reverse Primer	Accession No.	Amplicon size (bp)
CD40	5′-AACAGCGCCTGTGAAAGTTGAACAGCGCCTGTGAAAGTTG -3′	5′- GAAACCTCTGTCGCCATCTG -3′	NM_214194	80
CD8A	5′-CGAGGAGGACCAAGGCTACTCGAGGAGGACCAAGGCTACT -3′	5′- ACGGACATGAAGTTGCTGAA -3′	NM_001001907	75
CTLA4	5′-GAAGTCTGTGCCGCGACATAGAAGTCTGTGCCGCGACATA -3′	5′- GGAGGTGCCAGTGCATGTAG -3′	NM_214149	75
FYN	5′-GTGCAAAGTTCCCCATTAAGGTGCAAAGTTCCCCATTAAG -3′	5′- TTCCAAAAGACCACACATCA -3′	NM_001080206	87
GAPDH	5′-GATGCCCCCATGTTTGTGATGATGCCCCCATGTTTGTGAT -3′	5′- CAGGAGGCATTGCTGACGAT -3′	NM_001206359	77
ICOS	5′-CACTAGTGCACAGCGCTCTTCACTAGTGCACAGCGCTCTT -3′	5′- GCACGCAAAGGCATCTAAAC -3′	NM_001044546	78
IFI30	5′-CTCCACCTGTCAACGTGAACCTCCACCTGTCAACGTGAAC -3′	5′- GGGAAGAGCTCTCGGACTAG -3′	NM_001131046	79
IFNA1	5′-CAGACCTTCCAGCTCTTCAGCAGACCTTCCAGCTCTTCAG -3′	5′- GATCCAGTCCAGTGCAGAAC -3′	NM_214393	85
IFN-ALPHA-17	5′-CAGACCTTCCAGCTCTTCAGCAGACCTTCCAGCTCTTCAG -3′	5′- GATCCAGTCCAGTGCAGAAC -3′	NM_001195375	85
IFNGR1	5′-CACCATTGTGTCTTGGGATTCACCATTGTGTCTTGGGATT -3′	5′- GTTGCGTCCTCGTAAGTCTT -3′	NM_001177907	81
IL10	5′-CCAAGCCTTGTCAGAGATGACCAAGCCTTGTCAGAGATGA -3′	5′- CCTTGATATCCTCCCCATCA -3′	NM_214041	80
IL13	5′-GCGCCCTCTGTTCTCACAAGGCGCCCTCTGTTCTCACAAG -3′	5′- CTGGGCCACTTCAATTTTGG -3′	NM_213803	80
JAK1	5′-GGGCAGTACGATTTGGTCAAGGGCAGTACGATTTGGTCAA -3′	5′- GCCCAGACACTCGTTCTCAA -3′	NM_214114	90
NCR1	5′-GCTCATGGAGGGACATACAGGCTCATGGAGGGACATACAG -3′	5′- CTTTGACCAGGAGCTTCACA -3′	NM_001123143	88
NFATC1	5′-TGCAGGACTCCAAGGTCGTTTGCAGGACTCCAAGGTCGTT -3′	5′- CACAGGTCTCCGTCGGTTTT -3′	NM_214161	88
NFATC2	5′-AGCAGAATGCCACAAACCAAAGCAGAATGCCACAAACCAA -3′	5′- CACGCCCGAGGGTAATACTT -3′	NM_001113452	80
OAS1	5′-AAACCCAACCCGGAAATCTAAAACCCAACCCGGAAATCTA -3′	5′- GGTGGAAAACTCGCCCTCTT -3′	NM_214303	75
SLA-2	5′-AGCCATGGTGACTGGAGTTGAGCCATGGTGACTGGAGTTG -3′	5′- TGCCTGCAGCCTGAGTGTAG -3′	NM_001113702	79
SLA-3	5′-GGTGGTGAAAAAGGAGGGAGCTAGAAAAAGGAGGGAGCTA -3′	5′- CTTGGTGAGGGACACATCAG -3′	NM_001097427	75
SLA-DOA	5′-CGGGTCAGTTCACCTACGATCGGGTCAGTTCACCTACGAT -3′	5′- ACAGCCTCCCTCTTCTTCAG -3′	NM_00118514	70
SLA-DOB	5′-CCACTGGCCTCATCAGGAATCCACTGGCCTCATCAGGAAT -3′	5′- CCCCAAGCTCAGGAGTCATT -3′	NM_001114064	75

SOCS1	5′-CGTCCTCCGCGATTACTTGACGTCCTCCGCGATTACTTGA -3′	5′- ATAAGGCGCCCCAGTTAATG -3′	NM_001204768	88
TGFB1	5′-GTGCGGCAGCTCTACATTGAGTGCGGCAGCTCTACATTGA -3′	5′- GGTAGCCCTTGGGTTCATGA -3′	NM_214015	70
TNF	5′-CCCAGAAGGAAGAGTTTCCACCCAGAAGGAAGAGTTTCCA -3′	5′- CGGGCTTATCTGAGGTTTGA -3′	NM_214022	90

Table S3. List of genes used for mRNA sequencing data analysis

Gene symbol	Fold change	Average of read count		product	transcript_id	IPA-mapping symbol
		Control	HuNoV			
BATF	2.525	33	79.4	basic leucine zipper ATF-like transcription factor	NM_001244571.1	BATF
CCR7	8.666	17.4	166	C-C motif chemokine receptor 7	NM_001001532.3	CCR7
CD40	2.408	210	504.4	CD40 molecule	NM_214194.1	CD40
CD40LG	1.695	40.2	68.3	CD40 ligand	NM_214126.1	CD40LG
CD46	-1.797	7673.8	4200	CD46 molecule	XM_021102197.1	CD46
CD69	1.973	27.2	50.6	CD69 molecule	NM_214091.2	CD69
CD8A	1.643	138.8	217.2	CD8a molecule	XM_005662394.3	CD8A
CD8B	7.181	3.4	33.1	CD8b molecule	NM_001348770.1	CD8B
CSF2	2.433	18.2	42.9	colony stimulating factor 2	NM_214118.2	CSF2
CTLA4	2.209	4.4	10.7	cytotoxic T-lymphocyte associated protein 4	NM_214149.1	CTLA4
CXCL12	2.314	1768.8	3742.9	C-X-C motif chemokine ligand 12	XM_005671244.3	CXCL12
CXCR4	2.564	137.2	327.7	C-X-C motif chemokine receptor 4	NM_213773.1	CXCR4
EOMES	1.669	28.2	42.6	eomesodermin	XM_003132081.4	EOMES
FYN	1.826	687.2	1195.9	FYN proto-oncogene	XM_021084366.1	FYN
GATA3	1.606	184.4	281.5	GATA binding protein 3	XM_021064071.1	GATA3
ICOS	2.539	52.2	129.5	inducible T-cell co-stimulator	NM_001044546.2	ICOS
ICOSLG	1.858	327.4	583.4	inducible T-cell co-stimulator ligand	XM_005657167.3	ICOSLG
IFI30	1.600	2688.6	3872.6	IFI30, lysosomal thiol reductase	NM_001131046.1	IFI30

Table S3. Continued

Gene symbol	Fold change	Average of read count		product	transcript_id	IPA-mapping symbol
		Control	HuNoV			
IFN-ALPHA-1	4.868	1.4	11	interferon-alpha-1	NM_001166311.1	IFNA1
IFN-ALPHA-10	8.271	0	8.3	interferon-alpha-10	NM_001166310.1	IFNA10
IFN-ALPHA-13	2.939	0.6	3.7	interferon-alpha-13	NM_001164843.1	IFNA13
IFN-ALPHA-14	2.783	0.6	3.5	interferon-alpha-14	NM_001166318.1	IFNA14
IFN-ALPHA-15	2.057	5	12.2	interferon-alpha-15	NM_001164845.1	IFNA15
IFN-ALPHA-16	1.773	2.2	4.6	interferon-alpha-16	NM_001164855.1	IFNA16
IFN-ALPHA-17	4.903	1.2	9.9	interferon-alpha-17	NM_001195375.1	IFNA17
IFN-ALPHA-4	-2.991	4	0.5	interferon-alpha-4	NM_001166319.1	IFNA4
IFN-ALPHA-5	1.330	0.4	0.8	interferon, alpha 5	NM_001164860.1	IFNA5
IFN-ALPHA-8	4.535	1.4	11	interferon-alpha-8	NM_001164849.1	IFNA8
IFN-ALPHA-9	3.641	0.6	5.1	interferon-alpha-9	NM_001195377.1	IFNA9
IFNAR1	-1.261	889.4	630.3	interferon alpha and beta receptor subunit 1	XM_021068507.1	IFNAR1
IFNAR2	1.186	476	536.2	interferon alpha and beta receptor subunit 2	XM_021068453.1	IFNAR2
IFNB1	1.002	0	0	interferon beta 1	NM_001003923.1	IFNB1
IFN-DELTA-1	-1.597	3.6	2	interferon-delta-1	NM_001002832.1	IFND1
IFN-DELTA-10	2.129	0.6	2.3	interferon-delta-10	NM_001165907.1	IFND10
IFN-DELTA-11	1.519	0.6	1.5	interferon-delta-11	NM_001164851.1	IFND11
IFN-DELTA-2	-1.791	23	11.5	interferon-delta-2	XM_021063028.1	IFND2
IFN-DELTA-3	6.648	0	6.5	interferon-delta-3	NM_001164853.1	IFND3
IFN-DELTA-4	2.149	0	1.3	interferon-delta-4	NM_001164846.1	IFND4
IFN-DELTA-5	2.272	0.4	2.1	interferon-delta-5	NM_001164854.1	IFND5
IFN-DELTA-6	3.691	0.2	3.9	interferon-delta-6	NM_001166312.1	IFND6
IFN-DELTA-7	1.021	1	0.9	interferon delta 7	NM_001164859.1	IFND7
IFN-DELTA-8	3.022	0.2	3.3	interferon-delta-8	NM_001164847.1	IFND8
IFN-DELTA-9	1.630	0.6	1.5	interferon-delta-9	NM_001164848.1	IFND9
IFNG	-1.504	1.6	0.7	interferon gamma	NM_213948.1	IFNG
IFNGR1	-1.657	2334	1323.5	interferon gamma receptor 1	XM_005659183.3	IFNGR1
IFNGR2	1.050	1938.6	1908.9	interferon gamma receptor 2	NM_001111258.1	IFNGR2
IFNLR1	-1.599	428.4	273.3	interferon lambda receptor 1	XM_021095536.1	IFNLR1
IFN-OMEGA-2	-1.092	0.6	0.5	interferon-omega-2	NM_001166322.1	IFNW2
IFN-OMEGA-3	5.944	0	5.8	interferon omega 3	NM_001164844.1	IFNW3
IFN-OMEGA-4	3.301	0.6	5.1	interferon omega 4	NM_001164852.1	IFNW4
IFN-OMEGA-5	1.000	0	0	interferon omega 7	NM_001130533.1	IFNW5
IFN-OMEGA-6	1.687	2	3.7	interferon omega 6	NM_001164850.1	IFNW6
IFN-OMEGA-7	3.521	0	3.1	interferon omega 7	NM_001130232.1	IFNW7

Table S3. Continued

Gene symbol	Fold change	Average of read count		product	transcript_id	IPA-mapping symbol
		Control	HuNoV			
IL10	2.157	26.4	54.5	interleukin 10	NM_214041.1	IL10
IL12A	2.028	0.6	2.4	interleukin 12A	NM_213993.1	IL12A
IL12B	1.975	3	8	interleukin 12B	NM_214013.1	IL12B
IL13	2.680	3.4	9.7	interleukin 13	XM_005661643.3	IL13
IL24	4.248	27.4	127.1	interleukin 24	XM_021063319.1	IL24
IL29	1.366	1.6	2.7	interleukin 29 (interferon, lambda 1)	NM_001142837.1	IL29
IL2RG	1.764	972	1645.9	interleukin 2 receptor subunit gamma	XM_021079497.1	IL2RG
IRAK4	-1.495	302.6	189.2	interleukin 1 receptor associated kinase 4	NM_001112693.1	IRAK4
ISG15	-1.224	444.8	314.3	ISG15 ubiquitin-like modifier	NM_001128469.2	ISG15
JAG2	1.900	86.8	151.4	jagged 2	XM_021081548.1	JAG2
JAK1	-1.513	6334.6	3764.1	Janus kinase 1	XM_021093532.1	JAK1
JAK2	-1.770	671	343.4	Janus kinase 2	XM_021082799.1	JAK2
LAG3	2.879	58	146	lymphocyte activating 3	NM_001105306.1	LAG3
LAT	2.414	319.4	777.9	linker for activation of T-cells	XM_005662061.3	LAT
LCK	1.565	223.6	344.6	LCK proto-oncogene, Src family tyrosine kinase	XM_021093252.1	LCK
MX1	-2.334	1846.6	661.9	MX dynamin like GTPase 1	NM_214061.2	MX1
MX2	-1.587	201	105.8	myxovirus (influenza virus) resistance 2	NM_001097416.1	MX2
NCR1	2.459	13.2	32	natural cytotoxicity triggering receptor 1	XM_005664831.3	NCR1
NCR2	2.709	29.4	74	natural cytotoxicity triggering receptor 2	XM_021098670.1	NCR2
NCR3	3.014	7.8	27.3	natural cytotoxicity triggering receptor 3	XM_013977630.2	NCR3
NFATC1	2.010	245.4	455	nuclear factor of activated T-cells 1	NM_214161.1	NFATC1
NFATC2	1.844	382.8	654.3	nuclear factor of activated T-cells 2	XM_013985461.2	NFATC2
NOTCH1	2.221	741.6	1600.6	notch 1	XM_021081037.1	NOTCH1
NOTCH2	1.543	1024.8	1493.9	notch 2	XM_021090691.1	NOTCH2
NOTCH3	3.458	620.2	1988.3	notch 3	XM_021083631.1	NOTCH3
NOTCH4	2.318	209.4	455.6	notch 4	NM_001123147.1	NOTCH4
OAS1	-1.855	9751.8	5126	2'-5'-oligoadenylate synthetase 1	XM_021073680.1	OAS1
OAS2	-1.138	5356.8	4718.8	2'-5'-oligoadenylate synthetase 2	XM_021072182.1	OAS2
PDCD1	3.899	16	71.1	programmed cell death 1	XM_021074630.1	PDCD1

SH3BP2	1.640	369.8	557.7	SH3 domain binding protein 2	XM_005666488.3	SH3BP2

Table S3. Continued

Gene symbol	Fold change	Average of read count		product	transcript_id	IPA-mapping symbol
		Control	HuNoV			
SLA-1	-1.536	55600.8	32373.5	MHC class I antigen 1	XM_021098437.1	HLA-A
SLA-2	-2.189	51146.8	20509.2	MHC class I antigen 2	XM_021097872.1	HLA-B
SLA-3	-2.952	26341.4	7500	MHC class I antigen 3	XM_021097849.1	HLA-C
SLA-5	-2.541	13942.8	4558.3	MHC class I antigen 5	NM_001114056.1	HLA-H
SLA-6	1.577	436.6	617.1	MHC class I antigen 6	XM_013977620.2	HLA-E
SLA-7	1.136	993.2	1020.2	MHC class I antigen 7	XM_021098282.1	HLA-F
SLA-8	-1.104	546.2	453.7	MHC class I antigen 8	XM_013977617.2	HLA-G
SLA-DMA	1.813	2060.6	3487.1	SLA-DM alpha chain	NM_001113705.1	HLA-DMA
SLA-DMB	1.823	1007.2	1667.2	MHC class II, DM beta	NM_001113707.1	HLA-DMB
SLA-DOA	2.242	233.8	482.2	major histocompatibility complex, class II, DO alpha	NM_001185143.1	HLA-DOA
SLA-DOB	1.584	102	164.7	MHC class II, DO beta	XM_021097880.1	HLA-DOB
SLA-DQA1	2.124	5990.2	11524.4	MHC class II histocompatibility antigen SLA-DQA	XM_005665844.3	HLA-DQA1
SLA-DQB1	1.637	4970.2	7350.8	SLA-DQ beta1 domain	NM_001113694.1	HLA-DQB1
SLA-DRA	1.815	28180	45108.6	MHC class II DR-alpha	NM_001113706.1	HLA-DRA
SLA-DRB1	1.643	11662.6	18222.1	MHC class II histocompatibility antigen SLA-DRB1	NM_001113695.1	HLA-DRB1
SOCS1	2.375	306.2	693.4	suppressor of cytokine signaling 1	XM_005662126.3	SOCS1
STAT1	-1.389	6088.4	3818.3	signal transducer and activator of transcription 1	XM_021074657.1	STAT1
TGFB1	2.336	1400.8	3140	transforming growth factor beta 1	XM_021093503.1	TGFB1
TLR4	1.685	137.2	209.2	toll like receptor 4	NM_001113039.2	TLR4
TLR9	1.539	994	1358.1	toll like receptor 9	NM_213958.1	TLR9
TNF	4.633	24.6	113.9	tumor necrosis factor	NM_214022.1	TNF
TNFRSF1B	1.605	730	1119.7	TNF receptor superfamily member 1B	XM_021093133.1	TNFRSF1B
TYK2	1.798	1610.6	2810	tyrosine kinase 2	XM_005654826.3	TYK2
TYROBP	1.882	1330.2	2247.7	TYRO protein tyrosine kinase binding protein	XM_005664506.3	TYROBP
VAV1	1.523	288.6	421.9	vav guanine nucleotide exchange factor 1	XM_021079171.1	VAV1

Table S4. The color scale index for representing the relative expression level. The gene expression levels were indicated by different colors ranging from blue (low) to red (high).

Fold	Color	RGB
>10		150.5.1
>7.5		200.7.2
>5		250.8.2
>3		253.52.47
>2		253.102.98
>1.75		254.152.148
>1.5		255.202.201
>1.25		255.230.229
1		255.255.255
<-1.25		232.229.255
<-1.5		205.205.205
<-1.75		152.162.254
<-2		88.104.254
<-3		39.59.253
<-5		2.25.244
<-7.5		1.14.137
<-10		1.9.83
